# Supplementary material for: Women with Autoimmune Thyroiditis Taking Levothyroxine During Pregnancy: Is Iodine Supplementation Needed?
Source: Nutrients. 2025 Jan 31;17(3):542. doi: 10.3390/nu17030542 (PMC11820718; doi:10.3390/nu17030542)
Supplement: Supplementary file 1 [file nutrients-17-00542-s001.zip › nutrients-3424743-supplementary.pdf]

Date of birth: ___/___/___

Last menstrual period: _________

Week ok pregnancy: __________

Place of birth:

- Italy

- Foreign Country

Education (highest degree completed):

- none
- primary school
- middle school
- high school
- university

Do you smoke?

- No
- Ex-smoker
- < 10 sig/day
- > 10 sig/day

Do you use iodized salt?

- No
- Occasionally
- Yes, regularly

If yes, how long?

- < 6 months
- 6-12 months
- 1-2 years
- > 2 years

Do you use pregnancy supplements? Which one and since when?

- No
- Occasionally
- Yes, regularly

From ___ week of pregnancy. Commercial name: _________________

EATING HABITS

Cow milk:

- I never drink milk
- Few times a week
- I drink a glass/cup of milk a day
- I drink 2 or more glasses/cups of milk a day

Yogurt:

- I never eat yogurt
- I eat a cup of yogurt at least once a week
- I eat a cup of yogurt several times a week

Cheese:

- I never eat cheese
- I eat cheese at least once a week
- I eat cheese several times a week

Beef:

- I never eat beef
- I eat beef at least once a week
- I eat beef several times a week

Chicken/turkey:

- I never eat chicken/turkey
- I eat chicken/turkey at least once a week
- I eat chicken/turkey several times a week

Eggs:

- I never eat eggs
- I eat eggs at least once a week
- I eat eggs several times a week

Fish:

- I never eat fish
- I eat fish at least once a week
- I eat fish several times a week
